# Characteristics and outcomes of community-based perinatal peer support: Protocol for a systematic review

**DOI:** 10.1371/journal.pone.0303277

**Published:** 2024-07-01

**Authors:** Grace Branjerdporn, Hayley Kimball, Reaksmey Pirotta, Nataya Branjerdporn, Taryn Collins, Genevieve Bowman, Kerri M. Gillespie

**Affiliations:** 1 Mater Health, Catherine’s House for Mothers, Babies and Families, South Brisbane, Queensland, Australia; 2 Mater Research Institute—University of Queensland, South Brisbane, Queensland, Australia; 3 Outback Futures, Woolloongabba, Queensland, Australia; University of Birmingham, UNITED KINGDOM

## Abstract

**Background:**

Mental health issues and parenting difficulties in the perinatal period are common, and have significant negative impacts on individuals, families, and broader society. Community-based peer support programs might be an effective adjunct to standard mental health interventions in perinatal mental health issues, specifically where low-cost interventions are required, or access to professional care is limited.

**Methods:**

A systematic review will be undertaken. Searches will be conducted on four electronic databases (Pubmed, Embase, Cinahl, and PsycINFO), using terms related to perinatal mental health and peer support. Literature will be screened by title and abstract and then by full text. Selected studies will be evaluated using the Quality Assessment with Diverse Studies (QuADS) tool. Data relevant to community-based perinatal peer support intervention characteristics and outcomes will be extracted, and synthesised narratively.

**Discussion:**

This review will contribute to the existing evidence about perinatal mental health peer support, by synthesising information about community-based interventions specifically. The findings will be used to inform the design, implementation, and evaluation of a community-based perinatal mental health peer support program in urban and rural/remote hospital and health services in Australia.

**Trial registration:**

Systematic review registration: CRD42023451568.

## Introduction

### Mental illness in the perinatal period

The perinatal period, early childhood, and adolescence all pose a number of unique and complex challenges that can impact parents’ physical and psychological wellbeing. Adjusting to parenthood, particularly in first time parents, can be physically demanding, as well as challenging to personal identity, self-confidence, family dynamics and relationships. An estimated 20% of mothers, and around 10% of fathers experience poor mental health in the perinatal period [1–3]. However, less than 25% of parents seek professional help, due to lack of referral and support, stigma or shame and embarrassment, failure to recognise a problem, and financial concerns, among other reasons [4].

While perinatal depression and anxiety are the most common presentations, women may also experience a range of other mental health conditions [2, 5]. If untreated, perinatal mental health issues can result in long-term emotional, social, and wellbeing impacts for parents, children, and families. Maternal mental illness in the postpartum period has been found to impact attachment, and contribute to developmental delays in motor function, language acquisition, cognitive skills, emotional self-regulation, and adaptive behaviour [6, 7]. In acute cases, perinatal mental illness can result in maternal suicide or infant death [2]. Early detection and treatment of psychological distress or mental illness is critical, and can be life-changing for a mother and her infant.

### Peer support interventions for mental illness

‘Peer support’ is defined broadly as social and emotional support, often coupled with practical support, delivered by people who have themselves experienced mental health challenges [8, 9]. Peer support generally involves one (or more) of six core strategies: peer-led self-help groups, internet support groups, peer-delivered services, peer-run or -operated services, peer partnerships, and peer employees in health/social services [8]. Regardless of the model, peer support is always delivered as an adjunct to standard mental health interventions [8]. The inclusion of a paid peer support model of care has been growing in popularity over the past decade, and has been acknowledged by the World Health Organisation as a cost-effective model to deliver rights-based and person-centred care [10].

Peer support interventions may be delivered in inpatient, outpatient, digital, or in community settings [8]. Community peer support interventions are particularly important in rural and remote regions where residents are geographically distant from health services. In women diagnosed with perinatal mental illness, rurality is correlated with significantly higher parenting stress and lower access to supports [11]. Professional mental health care may also be financially prohibitive or simply unavailable for many, particularly those in developing nations. Community peer support interventions may help to address these gaps and their impacts.

In scoping searches, several systematic reviews and meta-analyses about peer support for perinatal mental illness were identified. These generally agree that peer support is effective at reducing self-reported and/or clinician scored symptoms of mental illness [12–14], and that it is considered by women to be important and useful [15, 16]. However, existing reviews are limited to specific conditions, such as perinatal depression [12, 14], specific models of peer support, such as internet-based interventions [16], or are restricted to qualitative studies or randomised controlled trials [12, 15]. Further, there are currently no systematic reviews identified which looked at peer support delivered in the community context specifically.

While much prior research has found peer support to be effective, empowering, and satisfactory for those receiving these services, a number of studies have found poorer outcomes [17], and identified a number of challenges to implementing successful peer support initiatives [18–20]. Challenges identified included lack of role clarity, feeling under-supported, difficulty maintaining boundaries, and judgemental peers [18–20]. An in-depth investigation of existing research will aid us in determining the backdrop to the emergence of these challenges and the model characteristics or strategies that may reduce or minimise any challenges or barriers. The current study will address the following aims:

1. Identify the characteristics of community-based perinatal peer support models of care described in the literature;
2. Evaluate the outcomes of community-based perinatal peer support models of care; and
3. Identify and classify how perinatal peer support models of care have been evaluated.

## Methods and analysis

We will complete a systematic review of the existing literature, including the current evidence and gaps in knowledge, on community-based perinatal peer support. This protocol describes the review methodology. It is structured using the Preferred Reporting Items for Systematic Review and Meta-Analysis Protocols (PRISMA-P) guidelines (S1 Checklist) [21]. The review is registered with the PROSPERO International Prospective Register of Systematic Reviews (Prospero ID CRD42023451568).

### Inclusion criteria

#### Types of studies

We will include all relevant quantitative and qualitative studies of primary data that investigate community-based perinatal peer support models of care. We will exclude systematic reviews, conference abstracts, meta-analyses, case studies, case series, dissertations, letters, poster abstracts, and scoping reviews or other literature reviews. Special populations such as perinatal loss, neonatal intensive care support, and breastfeeding are excluded. Studies will be limited to those published in English. We understand that this may lead to international studies being overlooked, and will discuss this in the limitations of our manuscript. See Table 1 for an overview of all inclusion and exclusion criteria.

**Table 1:** Inclusion and exclusion criteria.

PICOS	Inclusion	Exclusion
Population	Any parent with or without mental health difficulties or parenting challenges during pregnancy or postpartum (within the first 1000 days).	Special needs or Neonatal Intensive Care Unit (NICU) parent-baby dyads.
Intervention	Community-based peer support Co-facilitated by both a peer worker and health professional or early child educator Includes face-to-face modalities Includes continuity of care (more than one visit)	Phone support only Online support only No continuity of care (one visit only)
Outcomes	Intervention characteristics Clinical outcomes of women (such as depression, mother-baby attachment, parenting confidence, quality of life) Women’s perceptions and experiences of perinatal peer support Socio-economic outcomes	Breastfeeding only
Study design	Any qualitative or quantitative study containing primary data (retrospective or prospective cohort studies, cross-sectional studies, randomised control trials) Published in English, between 2012 and 2023.	Systematic reviews and scoping reviews Literature reviews Conference abstracts or posters Letters to the editor Commentary or opinions papers.

### Information sources

The searches will be undertaken on the following electronic databases: PubMed, CINAHL Complete (via Ebscohost), Embase, and PsycInfo (via Ovid). Searches will be conducted on titles and abstracts. Reference lists of identified papers and of any completed systematic reviews on similar topics would also be searched to ensure no papers were disregarded. All references will be exported into Covidence for the purposes of screening.

### Search strategy

Search terms and strategy were developed with all team members. The searches will use two groups of keywords, related to: (a) perinatal mental illness, and (b) peer support. The keywords will be truncated where needed, and MeSH terms will be used as appropriate, depending on database requirements. The terms will be combined using Boolean operators and parentheses. The search strategy is: (ante*natal OR antenatal OR ante*partum OR antepartum OR maternal OR peri*natal OR perinatal OR peri*partum OR peripartum OR post*natal OR postnatal OR post*partum OR postpartum OR pre*natal OR prenatal OR pregnan* OR parental OR birth OR “new parent” OR "perinatal care"[MeSH Terms]) AND (“peer* support” OR peer* OR “peer support*” OR “peer counsel*” OR “peer mentor*” OR “peer work*” OR “lived experience” OR befriender OR mentor). See S1 Table for specific search terms used.

### Screening

The search results will be exported into EndNote, and then into Covidence. Duplicates will be automatically removed by Covidence. Each study will then be screened against the eligibility criteria by a minimum of two reviewers to reduce the risk of bias. Studies will be screened by reading the title and abstract, then by reading the full text. Any disagreement between reviewers will be resolved by a third reviewer. The selection process will be documented in a Preferred Reporting Items for Systematic Reviews and Meta-Analyses (PRISMA) diagram [22].

### Data extraction

An electronic data extraction tool will be developed. Extraction will be completed by two independent reviewers. Data extracted will include data relating to the study (authors, dates, region, methods, sample size); the intervention (size and scope, peer and peer worker identification and training, strategies to match peers and peer workers, setting, frequency, duration of contact); and outcomes (clinical, social, and economic).

### Quality assessment

The studies selected for inclusion will be assessed using the Quality Assessment with Diverse Studies (QuADS) tool. This is a single tool shown to be reliable at assessing the quality of different study designs, across a variety of health- and social care-focused disciplines [23, 24]. Using this tool, studies will be judged to be at high, moderate, or low risk of bias.

### Data synthesis

Scoping searches show that studies about perinatal mental health peer support are highly heterogeneous–for example: they use a variety of study designs, test a mixture of outcomes, and report results in a range of ways. For this reason, a narrative synthesis will be undertaken. Data in relation to each of the outcomes will be organised thematically, critically examined for similarities and differences, and objectively reported using written text [25].

### Ethics

This systematic review does not require approval from a human research ethics committee.

## Discussion

Perinatal mental illness is common, and has significant detrimental impacts on individuals, families, and the wider community. Community-based perinatal mental health peer support might be an effective adjunct to standard mental health interventions, particularly where rurality impacts access to services, or where regular professional healthcare is financial prohibitive. This review will contribute to the existing evidence about perinatal mental health peer support, by synthesising the characteristics and outcomes of community-based perinatal mental health peer support.

The limitations of this systematic review must be acknowledged. The review only looks at literature indexed on a limited number of databases, and published in English; therefore, it is possible that other relevant studies may be overlooked. The authors will develop their own definition of ‘community-based’ peer support interventions, as none exist in the literature.

## Supporting information

S1 Checklist PRISMA-P 2015 checklist. (PDF)

S1 Table Search strategy. (DOCX)
