# Supplementary material for: Characteristics and outcomes of community-based perinatal peer support: Protocol for a systematic review
Source: PLoS One. 2024 Jul 1;19(7):e0303277. doi: 10.1371/journal.pone.0303277 (PMC11216617; doi:10.1371/journal.pone.0303277)
Supplement: S1 Table — (DOCX) [file pone.0303277.s002.docx]

**S2 Table: Search strategy**

| **Database** | **Search terms** |
| --- | --- |
| PubMed | (((((((((((((((((("ante*natal"[Title/Abstract] ) OR (antenatal[Title/Abstract])) OR ("ante*partum"[Title/Abstract])) OR (antepartum[Title/Abstract])) OR (maternal[Title/Abstract])) OR ("peri*natal"[Title/Abstract])) OR (perinatal[Title/Abstract])) OR ("peri*partum"[Title/Abstract])) OR (peripartum[Title/Abstract])) OR ("post*natal"[Title/Abstract])) OR (postnatal[Title/Abstract])) OR ("post*partum"[Title/Abstract])) OR (postpartum[Title/Abstract])) OR ("pre*natal"[Title/Abstract])) OR (prenatal[Title/Abstract])) OR (pregnan*[Title/Abstract])) OR (parental[Title/Abstract])) OR (birth[Title/Abstract])) OR ("new parent"[Title/Abstract])  OR  "perinatal care"[MeSH Terms]  AND  (((((((((((peer*[Title/Abstract]) OR ("peer* support*"[Title/Abstract])) OR ("peer support*"[Title/Abstract])) OR ("peer counsel*"[Title/Abstract])) ) OR ("peer* mentor*"[Title/Abstract])) OR ("peer mentor*"[Title/Abstract])) OR ("peer work*"[Title/Abstract])) OR ("peer* work*"[Title/Abstract])) OR ("lived experience*"[Title/Abstract])) OR (befriender[Title/Abstract])) OR (mentor*[Title/Abstract]) |
| Embase | antenatal:ab,ti OR ante*partum:ab,ti OR antepartum:ab,ti OR maternal:ab,ti OR 'peri*natal':ab,ti OR perinatal:ab,ti OR 'peri*partum':ab,ti OR peripartum:ab,ti OR post*natal:ab,ti OR postnatal:ab,ti OR 'post*partum':ab,ti OR postpartum:ab,ti OR 'pre*natal':ab,ti OR prenatal:ab,ti OR pregnan*:ab,ti OR parental OR:ab,ti OR birth:ab,ti OR "new parent":ab,ti  OR  'perinatal care'/exp  AND  peer*:ab,ti OR 'peer* support*':ab,ti OR 'peer support*':ab,ti OR 'peer counsel*':ab,ti OR 'peer* counsel*':ab,ti OR 'peer* mentor*':ab,ti OR 'peer mentor*':ab,ti OR 'peer* work*':ab,ti OR 'peer work*':ab,ti OR 'lived experience*':ab,ti OR befriender:ab,ti OR mentor*:ab,ti |
| Cinahl | MH perinatal care  OR  TI antenatal OR ante*partum OR antepartum OR maternal OR 'peri*natal' OR perinatal OR 'peri*partum' OR peripartum OR post*natal OR postnatal OR 'post*partum' OR postpartum OR 'pre*natal' OR prenatal OR pregnan* OR parental OR OR birth OR "new parent"  OR  AB antenatal OR ante*partum OR antepartum OR maternal OR 'peri*natal' OR perinatal OR 'peri*partum' OR peripartum OR post*natal OR postnatal OR 'post*partum' OR postpartum OR 'pre*natal' OR prenatal OR pregnan* OR parental OR OR birth OR "new parent"  AND  TI peer* OR 'peer* support*' OR 'peer support*' OR 'peer counsel*' OR 'peer* counsel*' OR 'peer* mentor*' OR 'peer mentor*' OR 'peer* work*' OR 'peer work*' OR 'lived experience*' OR befriender OR mentor*  OR  AB peer* OR 'peer* support*' OR 'peer support*' OR 'peer counsel*' OR 'peer* counsel*' OR 'peer* mentor*' OR 'peer mentor*' OR 'peer* work*' OR 'peer work*' OR 'lived experience*' OR befriender OR mentor* |
| PsycINFO | MA perinatal care  OR  TI antenatal OR ante*partum OR antepartum OR maternal OR 'peri*natal' OR perinatal OR 'peri*partum' OR peripartum OR post*natal OR postnatal OR 'post*partum' OR postpartum OR 'pre*natal' OR prenatal OR pregnan* OR parental OR OR birth OR "new parent"  OR  AB antenatal OR ante*partum OR antepartum OR maternal OR 'peri*natal' OR perinatal OR 'peri*partum' OR peripartum OR post*natal OR postnatal OR 'post*partum' OR postpartum OR 'pre*natal' OR prenatal OR pregnan* OR parental OR OR birth OR "new parent"  AND  TI peer* OR 'peer* support*' OR 'peer support*' OR 'peer counsel*' OR 'peer* counsel*' OR 'peer* mentor*' OR 'peer mentor*' OR 'peer* work*' OR 'peer work*' OR 'lived experience*' OR befriender OR mentor*  OR  AB peer* OR 'peer* support*' OR 'peer support*' OR 'peer counsel*' OR 'peer* counsel*' OR 'peer* mentor*' OR 'peer mentor*' OR 'peer* work*' OR 'peer work*' OR 'lived experience*' OR befriender OR mentor* |
